# Epidemiological survey on third molar agenesis and facial pattern among adolescents requiring orthodontic treatment

**DOI:** 10.4317/jced.53947

**Published:** 2017-09-01

**Authors:** Rafael Gómez de Diego, Javier Montero, Nansi López-Valverde, José Ignacio de Nieves, Juan-Carlos Prados-Frutos, Antonio López-Valverde

**Affiliations:** 1 Tenured Lecturer. Universidad Alfonso X El Sabio, Departamento de Estomatología. Villanueva de la Cañada, Madrid, Spain; 2 Tenured Lecturer. Universidad de Salamanca, Departamento de Cirugía. C/Alfonso X El Sabio, Salamanca, Spain; 3 Student, Universidad de Salamanca, Departamento de Cirugía. C/Alfonso X El Sabio, Salamanca, Spain; 4 Associate Professor, Universidad Alfonso X El Sabio, Departamento de Estomatología. Villanueva de la Cañada, Madrid, Spain; 5 Tenured Lecturer, Universidad Rey Juan Carlos, Departamento de Estomatología. Pozuelo de Alarcón, Madrid, Spain; 6 Tenured Lecturer, Universidad de Salamanca, Departamento de Cirugía. C/Alfonso X El Sabio, Salamanca, Spain

## Abstract

**Background:**

The aim of this study was to determine the association between facial pattern according to Ricketts cephalometric analysis, and prevalence of third molar agenesis, taking subject age and gender as control variables.

**Material and Methods:**

An epidemiological survey was conducted based on a sample of 224 candidates for orthodontic treatment aged 12 to 24 (n=224). Third molar agenesis was recorded using Ricketts cephalometric analyses of lateral teleradiographs and panoramic radiographs. The risk for agenesis was predicted considering the 5 Vert Index parameters (facial axis, facial depth, mandibular plane angle, lower facial height and mandibular arch), facial type (brachyfacial, mesofacial, dolichofacial) and sociodemographic variables (age and sex), using odds ratio (OR) calculated by logistic regression.

**Results:**

Third molar agenesis was observed in 25% of the sample. Risk for agenesis is significantly determined by sociodemographic factors (age, OR: 1.2), cephalic patterns (mesofacial vs dolichofacial, OR:4.3; and brachyfacial vs dolichofacial OR: 3.2) and cephalometric patterns (facial axis, OR: 0.8; lower facial height, OR: 0.8; and mandibular plane angle, OR:0.9).

**Conclusions:**

Facial parameters (facial axis, lower facial height, and mandibular plane angle) proved to be strong predictors of the risk for third molar agenesis, the prevalence of agenesis being significantly lower in dolichofacial individuals.

** Key words:**Facial Pattern, Ricketts Analysis, Third Molar Agenesis.

## Introduction

Tooth agenesis may affect any tooth, the third molar being the most frequently affected by this anomaly (5.3-56.0% range) (1-3). Environmental and genetic factors seem to play an etiological role in its occurrence (1,2). Some of the causes proposed involve disruption of the dental lamina, space limitation, abnormalities arising during embryogenesis, infection, trauma or massive exposure to ionizing radiation (1,2). Among the genetic factors are the trend towards smaller and fewer teeth or the progressive degeneration of craniofacial development (4,5), an opinion that is shared by certain anthropologists after observing that third molars do not always erupt as they used to in more primitive populations (6). Other authors are of the opinion that it is the result of a mutation and selection process based on genetic inheritance (7-9). Thus, tooth development is under a control that defines the position, number and shape of teeth, concluding that its aetiology is associated with an X-linked autosomal dominant or autosomal recessive pattern (8).

Agenesis can occur as an isolated condition (non-syndromic), or as part of a group of syndromes (syndromic) as in ectodermal dysplasia, Wiktop syndrome, or Rieger syndrome type I (9). This division means that one same genetic mutation can result in different phenotypic manifestations in different individuals, ranging from agenesis of one single tooth to complex syndromes (7,10). Cytogenetics and molecular biology are involved in the identification of the genes responsible for agenesis, establishing an underlying genotype-phenotype correlation. Recent findings seem to indicate that the ultimate cause of agenesis is genetic (7,8), as is the cephalic pattern.

Cephalometric analysis relates cranial shape to ethnography. The first attempts (gnathostatics) used photographic analyses to establish an association between teeth and bone structure. The introduction of skull lateral teleradiography overcame its limitations by studying craniofacial growth patterns, assessing dentofacial proportions and revealing the maxillary bases of malocclusion (11).

Ricketts cephalometric analysis uses 11 factors that locate maxillaries in space, situate denture in the face, and assess profile, designing three classifications (11,12): brachycephalic, mesocephalic and dolicocephalic.

**Figure 1:**
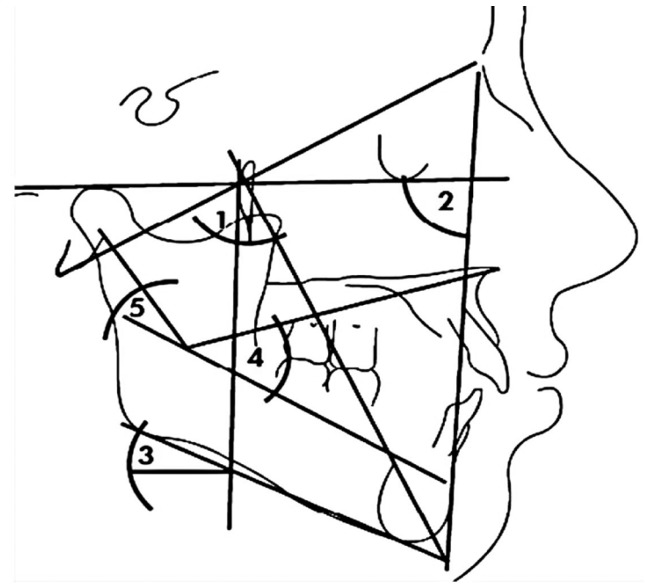
Facial pattern. 1) Facial depth angle. 2) Mandibular plane angle. 3) Lower facial height. 4) Mandibular arch.

Facial pattern, an identifiable trait, often provides a visual vertical description of the face. It is determined by a calculation involving five angles: facial axis, facial angle, mandibular plane angle, lower facial height and mandibular arch (Fig. 1). According to Ricketts (11,12), in normal distribution, 70% of the values score ± 1 standard deviation from the mean (mesofacial pattern), around 12.5% fall to the brachy or dolichofacial sides scoring ± 2 further standard deviation, the remaining 2.5% (extreme brachyfacial or dolichofacial cases) scoring above ± 2 standard deviation from the mean. Ricketts analysis also allows the use of the “Vert Index” numerical analysis that relates the five facial angles to a mathematical formula. Thus, the higher the negative value, the more dolichofacial the patient, while high positive values indicate strongly brachyfacial subjects. The values of “Vert Index” classify subjects into 6 types: severe dolichofacial, dolichofacial, mild dolichofacial, mesofacial, brachyfacial and severe brachyfacial (11,12).

In the literature available there are no conclusions as to a clear association between third molar agenesis and cephalometric measures; nevertheless, it has been suggested that third molar agenesis could be associated with horizontal, rather than vertical, cep-halometric measurements (13-18). Since the formation of the facial skeleton and dentition are conditioned by genetic factors that shape the resulting anatomical features, the main purpose of this survey was to study the relationship between third molar agenesis, analysing panoramic radiographies and facial pattern, through Ricketts cephalometric analysis.

## Material and Methods

Subjects (or their legal guardians, where appropriate) were fully aware of the purposes of the research and its methods. The study was carried out in conformity with the ethical principles and guidelines for research involving human subjects and with the approval of the Ethics Committee of the Faculty of Health Sciences of the Alfonso X El Sabio University of Madrid, granted on 1 September, 2015. The signed consent of all participants (or their legal tutors) was obtained prior to initiating the study.

The study population was obtained through consecutive sampling of orthodontic patients from a private dental clinic in Madrid (Spain), who had previously undergone a full pre-treatment study.

The inclusion criteria were.

• Age range between 12 and 24 years-of-age.

• Presence of the definitive second molar with fully erupted crown.

• No previous orthodontic treatment.

• No congenital diseases involving craniofacial abnormalities.

• Availability of a lateral cranial teleradiography and a panoramic radiography with sufficient quality to be subjected to cephalometric analysis.

Cases were defined on the basis of meeting the criteria and presenting agenesis of at least one third molar (using radiographic observation of the presence of tooth buds at different stages of mineralization). Cases were confirmed by the analysis of a panoramic radiography where the definitive second molar was at Demirjian’s seventh formation stage (19).

The controls were subjects with the same characteristics as those included in the study, without agenesis of the third molars.

The sample size needed was calculated using EPIDAT 3.1 epidemiological data analysis software in a pilot study with 100 candidates, the result being that a sample size of 224 subjects would be sufficient to establish 80% power statistically significant results among cephalic groups. 406 medical histories were reviewed, 227 of which fulfilled the study’s inclusion criteria, 59 with agenesis of at least one molar (cases) and 168 with no agenesis (control).

Third tooth agenesis (yes/no) was taken as the main dichotomous variable, although the number of missing teeth (from 0 to 4) and their position on the arcade was also recorded. The main independent variable was each subject’s facial pattern, expressed through the quantitative variable “Vert Index” (11) and the qualitative variable “facial pattern”, obtained from the “Vert Index”. Other independent variables included were the five angles of Ricketts cephalometric analysis (11) used to calculate “Vert Index” (these angles being facial axis, facial depth, mandibular plane angle, lower facial height and mandibular arch, as shown in Figure 1) and the sociodemographic variables “gender” and “age”.

SLTs were digitalised and imported to an HP laptop with NEMOCEPH 4.0 software (Nemotec Software, Madrid). The SLTs were calibrated and tracings from Ricketts cephalometric analyses were made. For SLTs that presented a split of anatomical structures, the intermediate point between both contours was used to locate the cephalometric landmarks. The programme calculated “Vert Index” based on each subject’s cephalometric characteristics.

The statistical analysis was carried out using SPSS v21 software for windows, setting the significance levels of *p*-values at < 0.05. Chi-square comparisons were used to compare the distribution of agenesis among facial groups and among quantitative variables. The Kolmogorov-Smirnov test was applied to check the normal distribution of the quantitative data (Z ranging from 0.78-0.98; *p*-values ranging from 0.31-0.64).

Afterwards Student T was used to compare the means of the cephalometric parameters among groups with agenesis and without it. Finally, a multivariate logistic regression analysis was conducted to predict the risk of third molar agenesis according to the sociodemographic and cephalic variables considered.

## Results

 Table 1 shows the sociodemographic and cephalometric descriptions of the study population (n=224). Subjects were aged from 12 to 24, with an average age of 15.5 ± 3.6, and there was a slight higher percentage of women (54%). Facial pattern analysis yielded a higher prevalence of brachyfacials (46.8%), mesofacials (18.7%) being the fewest. An analysis of the different degree of severity of facial patterns revealed a predominance of severe brachyfacials (34.3%), mild dolichofacials being the smallest group (12%). The relation between different age groups and facial patterns yielded a higher proportion of brachyfacial subjects in all three groups, followed by dolichofacial and mesofacial subjects. No significant differences were found when relating facial pattern to sex (*p*=0.5) or age (*p*=0.6).

**Table 1:**
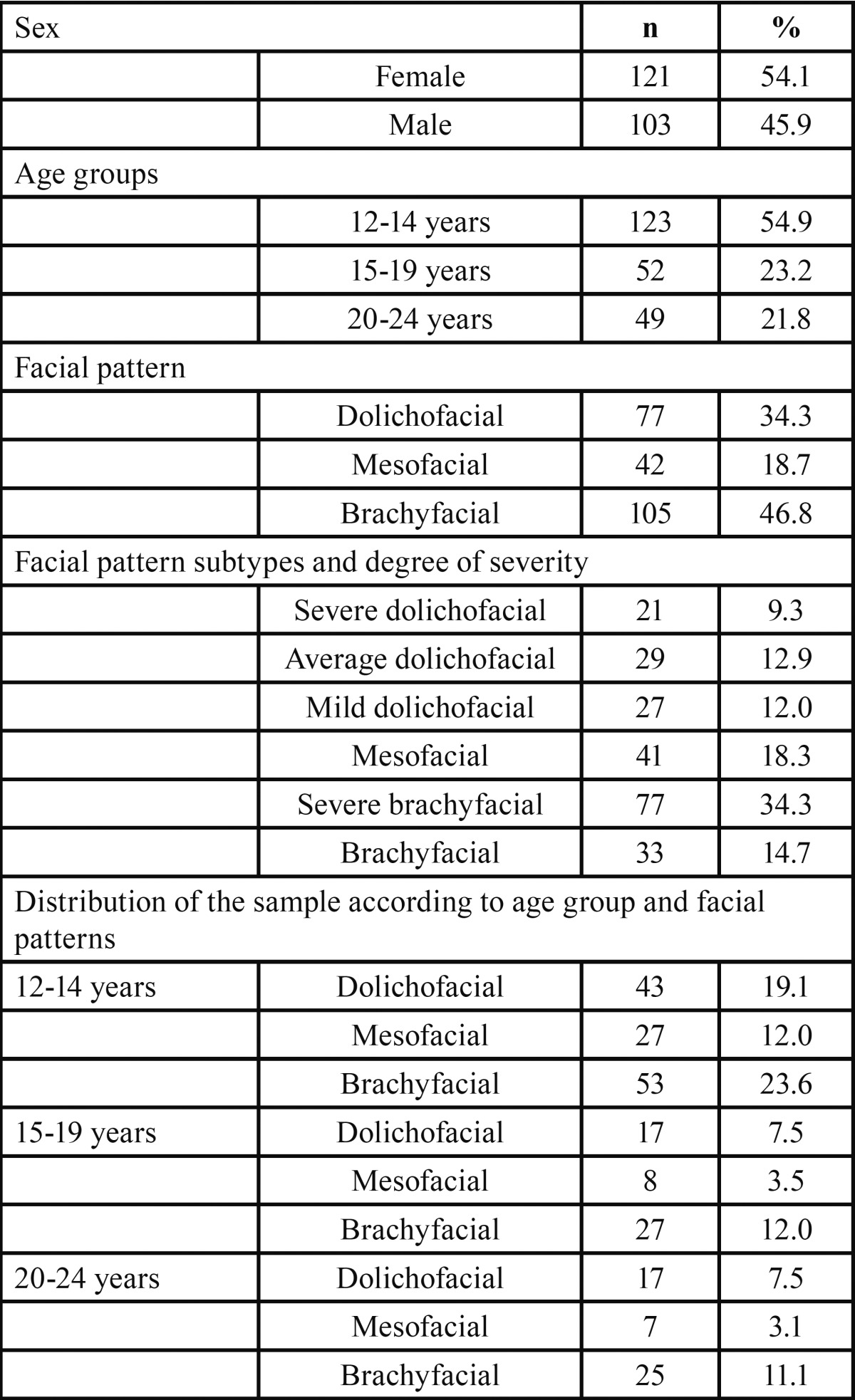
Sociodemographic and cephalometric description of the sample.

 Table 2 shows the prevalence and typology of tooth agenesis in the study population. Agenesis of one or more third molars was observed in 25%, with a higher, although not significant, frequency in women (28.1%). In terms of age groups, the prevalence of agenesis was higher in subjects between the ages of 15 to 19 (32.7%), than in the youngest age group (20.3%), although no statistical significance was observed. A total of 133 missing third molars was identified, the average in the cases being 2.5 ± 1.2 missing teeth. Distribution showed a prevalence of 2 missing third molars (10.3%), followed by 4 (7.6%), 1 (5.8%), and 3 third molars (1.3%). Frequency of occurrence was higher in mesocephalic (38.1%) than in dolichocephalic (14.3%) subjects.

**Table 2:**
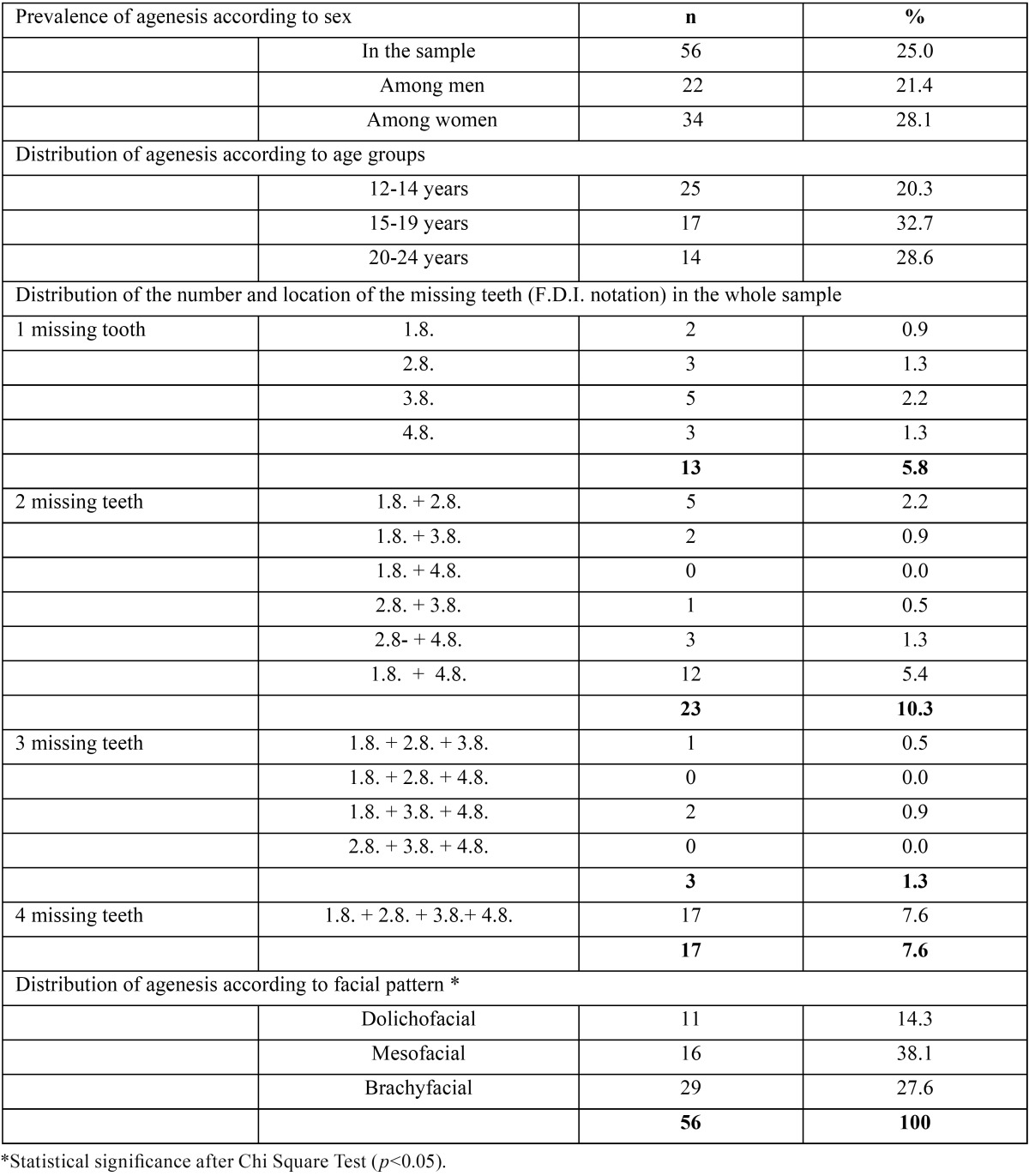
Prevalence and types of tooth agenesis in the study sample (n=224).

Moreover, subjects with agenesis showed a significantly shorter lower facial height (42.7 ± 4.9º) than those who were not affected by it (45.0 ± 5.4º), which is comparable for the rest of cephalometric values ( Table 3).

**Table 3:**
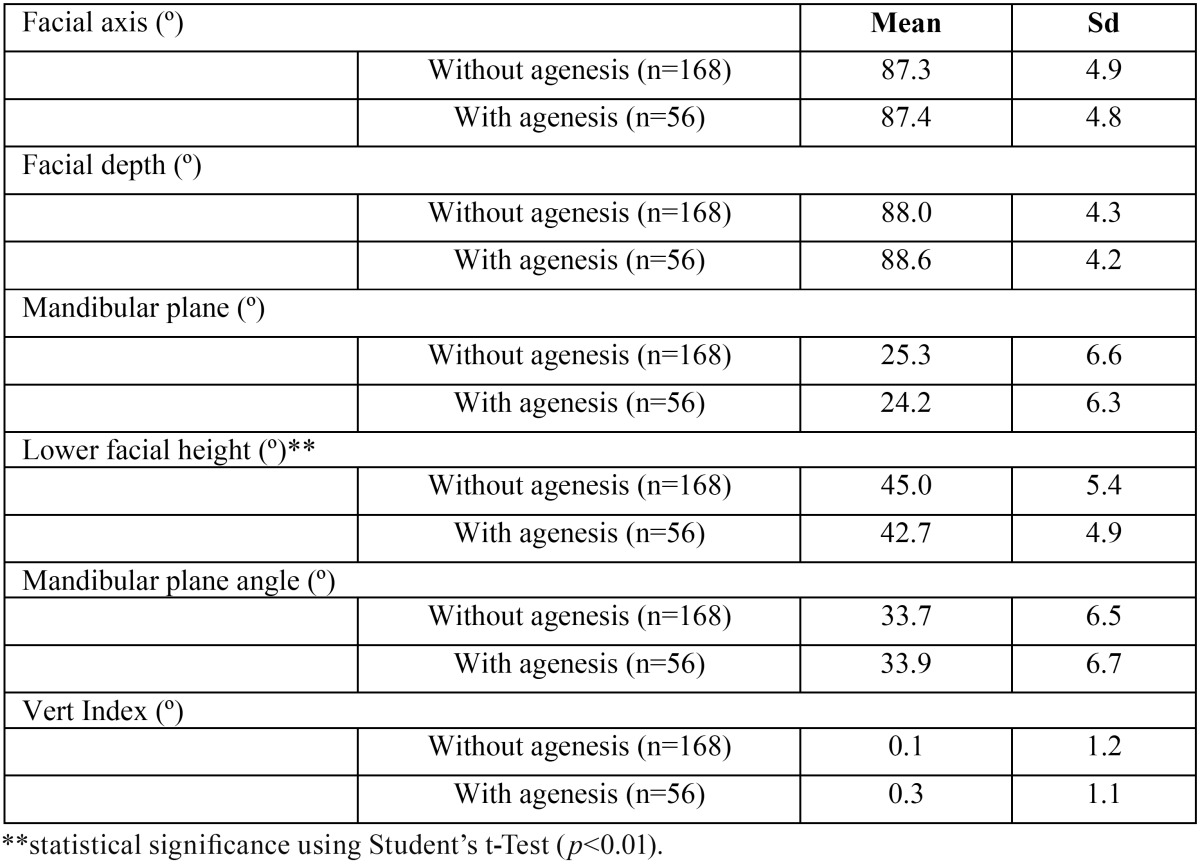
Relation between agenesis and Ricketts angular values (16).

There seem to be sociodemographic, cephalic and cephalometric factors that significantly predict the risk for agenesis ( Table 4). According to this model, risk is proportional to age [OR(95% CI): 1.2(1.0-1.3)], and mesocephalic [OR(95% CI): 4.3(1.5-12.4)] and brachycephalic [OR(95% CI): 3.2 (1.0-11.0)] subjects were at higher risk than dolychophalic individuals. Thus, the higher the values of the facial axis, lower facial height and mandibular plane angle, the lower the risk for agenesis.

**Table 4:**
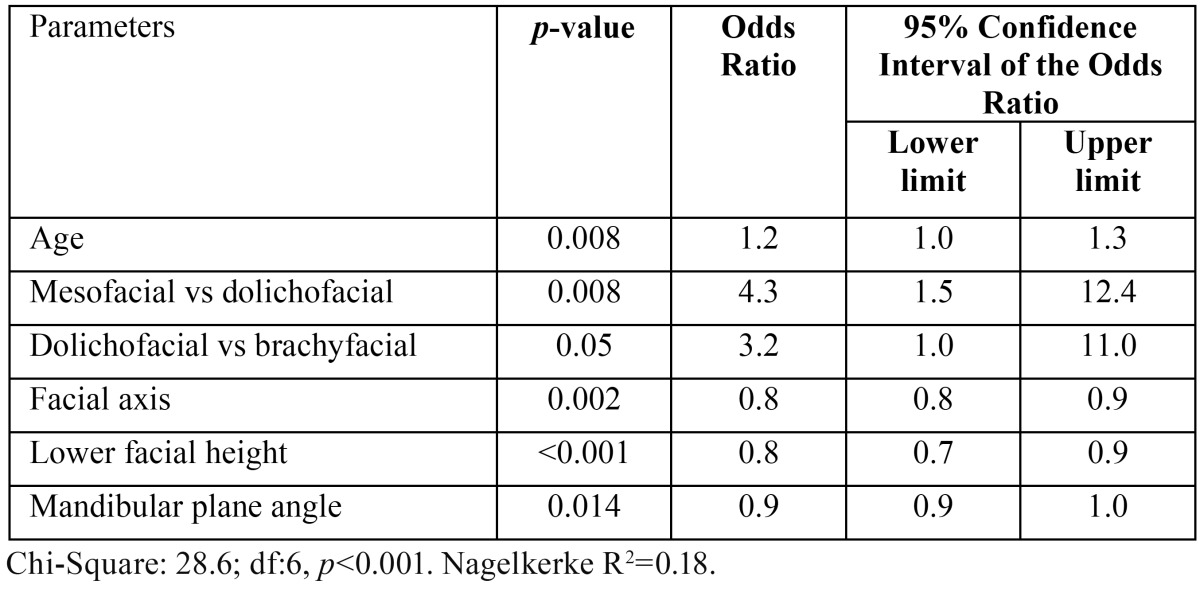
Relation between agenesis and Ricketts angular values (16).

## Discussion

Unlike most studies that examine craniofacial morphology from the horizontal perspective, this study examined the relationship between third molar agenesis in relation to vertical facial pattern (13-18). For this purpose, the advantage of using “Vert Index” as an instrument to measure vertical facial pattern is that it makes it possible to objectify the vertical facial pattern in a valid, easy and intuitive way (Fig. 1).

Based on the data obtained from the multivariate logistic regression analysis ( Table 4), agenesis appeared to be more prevalent in the brachyfacial pattern than in the dolichofacial [OR(95% CI): 3.2(1.0-11.0)], after controlling the rest of modulator variables. Sánchez *et al.*, (20) reported similar results from an analysis of the relationship between third molar agenesis and sagittal and vertical craniofacial morphology in a sample of 97 subjects divided into 3 groups, which showed a trend towards brachycephaly in subjects with third molar agenesis. On the other hand, Ben-Bassat and Brin (21) describe how a sample of infant population with a high number of missing teeth showed a trend towards mandibular retrognathism and low chin angle values, suggesting that it is due to tooth adaptation rather than to skeletal changes. Ogaard and Krogstad (22) reach similar conclusions after analysing 4 groups of 12-year-old subjects, observing lower anterior facial height reduction in those with severe hypodontia and concluding that such anomaly could be the result of functional compensation rather than skeletal traits. Conversely, other authors find no correlation between tooth agenesis and craniofacial structure. Thus, Yüksel and Üçem (17) found no significance between subjects with agenesis (n=74) and the control group (n=13), when correlating them with skeletal measurements.

Regarding age ( Table 4), it shows high significance (*p*=0.008) as a variable proportional to risk for agenesis, which would increase between 1.0 and 1.3 for every year of age reached. And this in spite of the fact that tooth buds would be more difficult to identify in the early stages of mineralization at an early age. Not all the studies conducted allow a comparison with the effect of age; Baba Kawano *et al.* (18), for example, do not include age as a variable, and Barka *et al.* (23) only analyse 13-year-old subjects. Kazanci *et al.* (24) set a minimum age limit of older than 15 for his study population. Similar differences can be noticed when comparing the work of Celiakaglu and Kamak (15) with that of Sandhu and Kahur (25): 17 years-of-age is the upper limit for the formers’ study, while being the lower age limit for the population analysed by the latter. However, the largest age discrepancy can be observed in Tavajohi-Kermani *et al.* (13) whose sample population includes different age groups ranging between 8 and 42 years-of-age. An analysis of this age diversity was recently conducted through a meta-analysis (26) of the prevalence of non-syndromic agenesis, concluding that the effect of ethnicity and continental land on the need for orthodontic treatment is not significant, against the significance of sample size and younger age groups. Thus, it is recommended that subjects under the age of 13 be left out of this type of studies.

With regard to sex, women showed a stronger trend towards agenesis ( Table 2), although it showed no significance in regression analysis ( Table 4). Results were similar to those obtained from a recent meta-analysis showing that women were more frequently (14%) (3). However, other studies do not gather any significant differences when relating gender and this anomaly (23,24). Thus, research by Levesque *et al.* (27), based on a sample of French-Canadian subjects (n=4640) in the 7 to 25 age range yields no significant differences. Kazanci *et al.* (24), report identical results using a sample of young Turkish subjects (n=2580) aged between 12 and 16. A recent study that analysed 220 panoramic radiographs from a group of orthodontic patients from northern Greece concludes that inter-sex differences in the number of third molars affected by agenesis are not significant (23).

When assessing the number of non-developed teeth, agenesis was most frequently associated with the absence of 2 third molars, followed by all 4, 1 and 3 ( Table 2). On the other hand, when Richardson (28) carried out a comparison between two groups of subjects, the results obtained showed delayed tooth bud development (cases) against early formation (control), 1 molar agenesis being observed as the most prevalent (41%) and 3 as the most infrequent (5.9%), with similar results for agenesis of 2 and 4 teeth. Kazanci *et al.* (24) report 1 third molar agenesis as the most frequent (9.2%), 3 being the rarest (2.6%). Although the mentioned results have a certain degree of inter-study variability, they show that agenesis affecting 1 or 2 teeth is the most common.

In terms of site-specificity of tooth agenesis with respect to the hemimaxillaries, it appeared more frequently on the left side than on the right, showing no significance. In this regard, most of the authors reviewed in the literature research carried out for the purpose of this study, found no significant differences when relating third molar agenesis and the hemimaxillary position of the affected teeth (3,14).

Dental arch analysis revealed that agenesis was more prevalent in the mandible (56.1%) as compared to the upper maxilla (43.9%). Richardson (28), reaches the conclusion that lower third molars are affected by agenesis more frequently than upper ones (55-31%).

No significance was observed when relating the variables “Vert Index” and third molar agenesis (*p*=0.21). A review of the literature shows disagreement when relating the dependent variable and cephalometric measures, being most frequently associated to the horizontal ones (13-17). In the study conducted by Sánchez *et al.* (20), the authors conclude that the group affected by mandibular third molar agenesis presents a diminished lower facial third and a brachyfacial pattern, whereas agenesis of maxillary third molars is associated with low mandibular plane angle values. The lack of significance observed is probably the result of using “Vert Index”, while Sánchez *et al.* (20) examine 10 variables associated with the vertical classification of the face, reporting significance of three of them. The results of our study showed high significance ( Table 3, Table 4) in the correlation between agenesis and lower facial height (*p*<0.001; OR:0,8). These results are similar to those reported by Sánchez *et al.* (20) (*p*=0.01) and by Gungor and Turkkahraman (29) (*p*<0.01), who compared facial height and congenital absence of teeth in a sample population (n=154) of subjects divided into cases and controls. Likewise, a review of the literature focused on age, prevalence and risk factors associated with congenital absence of teeth, reports the association between vertical skeletal changes and tooth agenesis in its three forms (hypodontia, oligodontia and anodontia), concluding that the shortening of lower facial height leads to more severe forms of agenesis, the front teeth being especially affected (30). Similar results were obtained by Ogaard and Krogstad (22) when comparing two study groups of subjects with (n=87) and without agenesis (n=50), concluding that there are very few skeletal variables, one of them being reduction of lower facial height, associated with the increase of missing teeth.

The mandibular plane angle indicates mandibular growth direction, taking high values in dolichofacial subjects and low values in brachyfacial ones (13). Logistic regression yielded significance when using this variable ( Table 4) as a potential predictor of the risk for agenesis (OR:0.9-1.0; *p*=0.01). This means that the wider the mandibular angle, the lower the risk for agenesis. Similar results were obtained by Ogaard and Krogstad (22), who found a relation between increased occurrence of agenesis and low mandibular plane angle values. In his review of the literature, Rakhshan (30) also states that the more acute the values of the mandi-bular plane angle, the more prevalent the occurrence of congenital agenesis of front teeth excluding third molars.

Facial axis, whose value depends on the shape and position of the mandible, indicates the direction of growth of the chin and remains practically unchanged by age. Thus, values above 90º point to forward growth (brachyfacial pattern). The results of the logistic regression carried out that show the high significance of the predictive capacity of this variable are gathered in  Table 4, where this value proves to be inversely proportional to the risk for agenesis (OR=0.8-0.9; *p*=0.002). The lower facial height variable showed a similar behaviour (OR=0.7-0.9; *p*<0.001). Several studies confirmed the relation between lower facial height and agenesis (4,17,20,22,29).

Against this background, it has not been possible to prove the predictive capacity of the summary “Vert Index”, not even with certain of its parameters (facial angle and mandibular arch), although evidence was found that the rest of its components (lower facial height, mandibular plane angle and facial axis) are significant predictors of the occurrence of third molar agenesis when oriented towards values characteristic of brachyfacial subjects. These findings confirm the initial hypothesis that this abnormality is potentially associated with certain vertical facial features. Future epidemiological surveys with larger sample sizes and different target populations are suggested to verify the validity of our findings.

## Conclusions

Facial parameters (facial axis, lower facial height and mandibular plane angle) proved to be significant predictors of the risk for third molar agenesis, this phenomenon being significantly less prevalent in dolichofacial than in brachyfacial and mesofacial individuals.
